# Tobacco Smoke Exposure Reduces Paraoxonase Activity in a Murine Model

**Published:** 2017-03

**Authors:** Robert M. Reed, Saif M. Borgan, Michael Eberlein, Monica Goldklang, Joshua Lewis, Michael Miller, Mohamad Navab, Bo S. Kim

**Affiliations:** 1 University of Maryland School of Medicine, Division of Pulmonary and Critical Care Medicine, 110 South Paca Street, 2^nd^ Floor, Baltimore, Maryland 21201, United States;; 2 University of Iowa School of Medicine, Division of Pulmonary and Critical Care Medicine, C326 GH Iowa City, Iowa 52242. United States;; 3 Division of Anesthesia, Columbia University, Anesthesiology, New York, New York, United States;; 4 Division of Endocrinology, Diabetes, and Nutrition, University of Maryland School of Medicine, Howard Hall, 498, Maryland, United States;; 5 Division of Cardiology, University of Maryland School of Medicine, 110 South Paca Street, Baltimore, Maryland 21201, United States;; 6 David Geffen School of Medicine, Division of Cardiology, Le Conte Ave , Los Angeles, California 90095, United States;; 7 Division of Pulmonary and Critical Care Medicine, Johns Hopkins University School of Medicine, 1830 E. Monument Street, Baltimore, Maryland 21287, United States

**Keywords:** Paraoxonase, High density lipoprotein, tobacco smoke, antioxidants

## Abstract

**AIM:**

To demonstrate a direct inhibitory effect of cigarette smoke exposure on paraoxonase 1 activity in a murine *in vivo* model.

**METHODS:**

At 8 weeks old, we randomized 10 C57/bl6 mice to an environment consisting of either filtered air or cigarette smoke for 6 months. Smoke exposure (7 hours per day, 5 days per week) was standardized using a model TE-10 smoking machine and adjusted to maintain constant sidestream and mainstream smoke. After 6 months of exposure, we assessed differences in lung air space, cholesterol, lipid, and lipoprotein profiles, as well as paraoxonase activity in mice exposed to cigarette smoke extract compared to unexposed control mice.

**RESULTS:**

Cigarette smoke exposure by the protocol used was sufficient to result in pathologic changes in lung architecture consistent with emphysema. Specifically, we observed that mice exposed to cigarette smoke had a significantly higher mean linear chord length compared to animals that were exposed to filtered air (*p*<0.02). Despite this exposure, no differences in total HDL-cholesterol levels or HDL-cholesterol sub-fractions (i.e. HDL2 and HDL3 fractions) were noted between smoke-exposed and unexposed animals (*p*=1.00, 0.6, and 0.4, respectively). Notably, mean HDL-cholesterol levels were identical between groups (92.8 vs 92.8 mg/dL, *p*=1.0). Paraoxonase activity, however, was markedly reduced in mice exposed to cigarette smoke compared to those who were not exposed (102, SD=9.6 vs 144, SD=4.1 units of activity, respectively, *p*=0.002).

**CONCLUSION:**

In this murine model, tobacco smoke exposure directly inhibits paraoxonase activity independently of HDL-cholesterol levels rather than indirectly via reduction in HDL-cholesterol levels.

## INTRODUCTION

In 1946, Mazur *et al* described an endogenous enzyme that is capable of inactivating a group of bioactive, potentially toxic chemicals known as organophosphates (1). This enzyme is now identified as paraoxonase (PON) and research is still underway to unravel the full spectrum of its properties, including its anti-oxidant, anti-inflammatory and anti-cholinergic actions (2). Three subclasses of the PON family have been discovered (PON1, PON2 and PON3), with variations in their function and potency. All PON enzymes share similar anti-oxidant properties, but unlike its counterparts, PON2 lacks any activity against organophosphate. The inhibitory action of PON1, and to a lesser extent PON3, on organophosphates was one of the first described functions of the PON family and is significant when considering the effects of organophosphates on the body. Organophosphates are a class of bioactive chemicals first described around 1850 by Philippe de Clermont, but not appreciated to be toxic until the 1930s (3). Organophosphate toxicity manifests through strong anticholinergic effects, and organophosphates are used in a variety of applications including as pesticides and as biological weapons such as sarin gas. Consistent with its *in vitro* ability to inactivate organophosphates, lower PON activity as well as genetic variants associated with lower PON activity are associated with increased susceptibility to organophosphate poisoning (4).

Paraoxonase 1 is a calcium-dependent enzyme produced primarily in the liver and circulates the body in association with high density lipoprotein (HDL) (2). PON1 is thought to exhibit various anti-atherosclerotic properties through inactivation of oxidized LDL, stimulation of HDL-mediated cholesterol efflux from macrophages, and suppression of monocyte transformation into foam cells (2, 5).

Several cross-sectional studies studies have shown tobacco smoking to correlate with lower PON activity (2, 6-9). In those studies, smoking was also associated with lower levels of HDL-cholesterol which in turn correlate strongly with PON1 activity. While statistical analyses indicated the reductions in HDL-cholesterol seemed at least partially independent, the possibility for unmeasured confounding remain. While *in vitro* studies clearly demonstrate PON1 inhibition by cigarette smoke extract directly, to our knowledge controlled *in vivo* studies are lacking.

Thus we sought to characterize PON levels and lipid profiles associated with tobacco smoke exposure in a murine model in order to evaluate the hypothesis that PON reductions *in vivo* occur primarily through direct effects with little indirect effect attributable to reduced HDL levels.

## MATERIALS AND METHODS

### Murine cigarette exposure

Smoke exposure was performed as previously described (10). In brief, 10 C57/bl6 mice were randomized at 8 weeks of age to receive either filtered air or cigarette smoke exposure for 7 hours a day, 5 days a week. Smoke was generated using Kentucky 3R4F cigarettes (Tobacco Research Institute, University of Kentucky, Lexington, KY, USA) in a model TE-10 smoking machine (Teague Enterprises, Woodland, CA, USA). Each cigarette was puffed for seconds every minute for a total of 8 puffs, at a flow rate of 1.05 L/min to generate a standard 35 mL puff. Exposure was adjusted to maintain a mixture of 89% sidestream smoke and 11% mainstream smoke. After 6 months of exposure, mice were removed from smoke for over 24 hours and then sacrificed to permit collection of serum and lung samples. Emphysematous changes were quantified by measuring mean linear chord length as an index of airspace size (11).

### Lipid profiles

Cholesterol levels were measured using the vertical auto profile method, which is a density gradient ultracentrifugation technique (12). Paraoxonase activity was measured as previously described (13, 14). Detailed methods for paraoxonase measures are included in methods supplement #1, #2.

### Statistical analysis

Data are presented as means (standard deviation). Comparisons between mice exposed to cigarette smoke and filtered air were made using Student t tests. Data were analyzed using STATA 14 SC software (StataCorp-LP, College Station, TX, USA). For all analyses, two-tailed p<0.05 was considered significant.

## RESULTS

**Figure 1:**
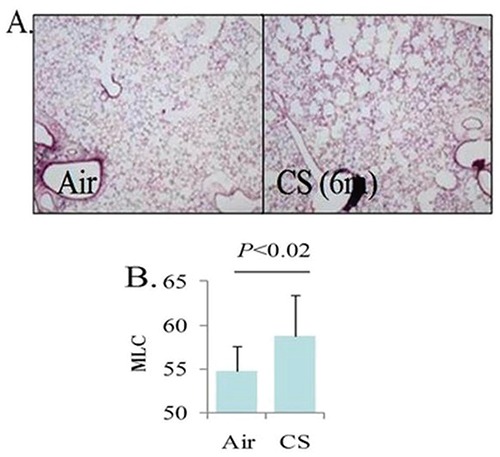
Microscopic appearance of lung tissue in mice exposed to filtered air versus cigarette smoke. Alveolar spaces are appreciably enlarged which is consistent with emphysematous changes; B) Mean linear chord (MLC) length provides a quantitative confirmation of emphysematous changes. CS, cigarette smoke.

**Figure 2:**
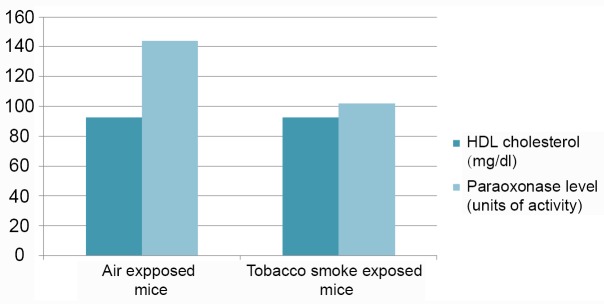
Effect of tobacco smoke exposure on HDL-cholesterol levels (dark) and paraoxonase activity (light) in a murine model. In the tobacco smoke exposed murine model (right side), paraoxonase activity was significantly reduced when compared to the air exposed model on the left (102 vs 144 units of activity, *p*-value =0.002). There were no noticeable differences in HDL cholesterol levels between the tobacco smoke exposed and the air exposed models (92.8 vs 92.8 mg/dl, *p*-value=1.0).

Consistent with previous reporting (10), cigarette smoke exposure by the protocol used was sufficient to result in pathologic changes in lung architecture consistent with emphysema (Figure 1). Specifically, we observed that mice exposed to cigarette smoke had a significantly higher mean linear chord length compared to animals that were exposed to filtered air (*p*<0.02). Importantly, however, no differences in total HDL-cholesterol levels or HDL-cholesterol sub-fractions (i.e. HDL2 and HDL3 fractions) were noted between smoke-exposed and unexposed animals (*p*=1.00, 0.6, and 0.4, respectively). In addition, serum LDL and very low-density lipoprotein (VLDL) levels were similar between intervention groups (*P*=0.09, 0.07, and 0.2 for levels of LDL, VLDL, and VLDL3, respectively). Complete analysis of lipid profiles between mice exposed to cigarette smoke and control mice are shown in Table 1. Notably, mean HDL-C levels were identical between groups (92.8 vs 92.8 mg/dL, *p*=1.0). Paraoxonase activity however, was significantly lower in the smoke exposed group compared to control animals (102 vs 144 units of activity, respectively, *p*=0.002) (Figure 2).

**Table 1:** Lipid profiles of mice exposed to either filtered air or cigarette tobacco smoke for 6 months

	Air	Tobacco Smoke	P

Total Cholesterol (mg/dL)	153.6 (15.4)	142.2 (116.3)	0.4
HDL Cholesterol (mg/dL)	92.8 (6.1)	92.8 (9.8)	1.0
Non-HDL Cholesterol (mg/dL)	61.0 (9.4)	49.4 (34.1)	0.1
HDL2 Cholesterol (mg/dL)	28.8 (4.5)	30.6 (6.9)	0.6
HDL3 Cholesterol (mg/dL)	64.0 (2.9)	62.2 (3.3)	0.4
Lp(a) Cholesterol (mg/dL)	16.4 (4.5)	11.4 (3.4)	0.08
Total LDL (LDL-R + Lp(a) + IDL Cholesterol) (mg/dL)	48.2 (9.3)	35.4 (11.5)	0.09
LDL-R (mg/dL)	34.4 (5.3)	26.4 (7.6)	0.09
VLDL Cholesterol (mg/dL)	12.8 (0.4)	14 (1.2)	0.07

HDL, high density lipoprotein; IDL, intermediate density lipoprotein; Lp, lipoprotein; LDL, low density lipoprotein; LDL-R, real low density lipoprotein. Data are expressed as mean (SD).

## DISCUSSION

In this investigation, we observed that mice exposed to cigarette smoke had markedly reduced paraoxonase activity compared to control mice despite the fact that mean HDL-cholesterol levels were identical.

Our findings indicate that the magnitude of direct effect on PON levels that cigarette exposure exerts seems considerably greater than the magnitude of effect the same exposure manifests on HDL-cholesterol levels. Notably, our data should not be taken to suggest that smoke exposure does not result in reduced HDL-cholesterol as this is well established in both humans as well as murine models (15-17). The lack of reduction in HDL-cholesterol levels was not expected and may be explained by inadequate power in our sample. Importantly, however, our findings do indicate that cigarette smoke exposure seems to directly inhibit PON1 activity independently of HDL-cholesterol levels. While previous investigations show that a reduction in HDL-cholesterol levels do lead to a concominant decrease in PON1 activity (2), the magnitude of direct effect on PON levels that cigarette exposure exerts seems considerably greater than the magnitude of effect attributable to tobacco smoke related reductions in HDL-cholesterol levels.

A less-likely alternate hypothesis as to why cigarette exposure failed to result in a reduced HDL-cholesterol level relates to the emphysematous changes observed. Studies primarily including former smokers have observed a correlation between emphysema and elevated levels of HDL-cholesterol (18-23). As such, it is difficult to exclude the possibility that the cigarette smoke related reduction in HDL-cholesterol levels was mitigated by the development of emphysema.

Since PON has anti-atherosclerotic effects, the direct inhibitory effects of tobacco smoke on PON activity, regardless of HDL level, may help to explain the excess cardiovascular disease (CVD) risk observed in chronic obstructive pulmonary disease (COPD). Several studies have suggested that HDL does not always reduce cardiovascular risk (24-26). This is thought due to periodic conversion of HDL to a pro-inflammatory state characterized by low PON1 activity (25-27).

The frequent coexistence of COPD and CVD is not fully explained by traditional risk factors including smoking and lipid levels (22, 28-37). The mechanisms of this association are unknown, and the association persists in patients who have never smoked (32, 37). A hypothesis that stems from our findings in context with the literature available is that PON activity may in large part explain this observation. Genetically determined variations in PON activity have been implicated in not only cardiovascular, but also pulmonary function (2). In addition to its role as an HDL-associated multifunctional antioxidant enzyme, PON1 is also found in non-ciliated bronchiolar epithelial (Clara) and type 1 alveolar cells of the lung as well as in the vascular endothelium. Data suggest that PON1 protects the airway epithelium as well as vascular endothelium through anti-oxidative and anti-inflammatory effects, and that it is integral to the cardio-protective properties of HDL. Reduced PON1 activity impairs the normal function of HDL to remove oxidized phospholipids. Such oxidized phospholipids facilitate the recruitment and activation of monocytes to the vascular intima (25, 27, 38, 39). HDL is also known to exert anti-inflammatory effects in the lung (40). However, since COPD has been associated with both, high HDL levels (18-23) and high rates of CVD (22, 28-37), this discrepancy could be explained by a dysfunctional HDL mechanism in this population. Our prior data suggest high HDL-cholesterol in this population does not associate with reduced cardiovascular disease (22), and reduced paraoxonase activity has been described in association with COPD (41). Notably, paraoxonase levels in smokers without COPD were not reduced (41) and are strongly affected by genetic polymorphisms (41, 42). These polymorphisms in turn have been implicated in susceptibility to COPD (41, 43) as well as cardiovascular disease (44).

The strength of our conclusions is limited by the sample size, which could not be increased due to the closure of the lab (Atherotec Diagnostics Lab) where lipid studies were performed. Consequently the robustness of the findings are limited and the results warrant replication.

## CONCLUSIONS

In a murine model, tobacco smoke exposure was associated with lower levels of paraoxonase activity despite no differences in HDL-cholesterol levels compared to control mice. This suggests that tobacco smoke exposure primarily acts directly to reduce paraoxonase levels rather than indirectly via reduction in HDL-cholesterol levels.

## COMMENTS

### Background

Paraoxonase 1 (PON1) is an enzyme that binds to high density lipoprotein (HDL) particles and imparts antioxidant and antiatherogenic properties. Cigarette smoke exposure is associated with reduced PON1 activity in cross-sectional correlative studies through what appears to be both direct effects as well as indirect effects as a result of a smoking related reduction in HDL particles. *In vivo* administration of cigarette smoke further supports a direct inhibitory effect. We sought to demonstrate a direct inhibitory effect of cigarette smoke exposure on PON1 activity in a murine in vitro model.

### Research frontiers

Lung function and vascular disease demonstrate correlation that is independent of common risk factors, such as tobacco smoke exposure. The mechanisms of that associated are incompletely understood and paraoxonase is a plausible target for future study.

### Innovations and breakthroughs

We demonstrated a reduction in paraoxonase activity associated with tobacco smoke exposure despite no differences in HDL-cholesterol levels in a murine model. This suggests that tobacco smoke exposure primarily acts directly to reduce paraoxonase levels rather than indirectly via reduction in HDL-cholesterol levels. Due to the small sample size, results warrant replication.

### Applications

As paraoxonase 1 has been correlated to both lung and vascular function, it represents a plausible intermediary warranting further study as a biomarker, and may pose a novel target for future therapeutics.
